# Internal embryonic development in a non-copulatory, egg-laying teleost, the three-spined stickleback, *Gasterosteus aculeatus*

**DOI:** 10.1038/s41598-019-38584-w

**Published:** 2019-02-20

**Authors:** Laura L. Dean, Shaun Robertson, Muayad Mahmud, Andrew D. C. MacColl

**Affiliations:** 1 School of Life Sciences, University of Nottingham, Nottingham, NG7 2RD UK; 2 Scientific Research Center, Erbil Polytechnic University, Erbil, Iraq

## Abstract

The switch from egg-laying to retaining and giving birth to live young is a major transition in the history of life. Despite its repeated evolution across the fishes, records of intermediate phenotypes are vanishingly rare, with only two known cases in existence of normally egg-laying fish species retaining embryos within the ovaries. We report the discovery of a third occurrence, in which well-developed embryos were found in the ovaries of a three-spined stickleback (*Gasterosteus aculeatus*), a non-copulatory, normally oviparous species. Extracted from the parent fish, these embryos hatched and grew to adulthood. Genetic and physiological examination of the parent fish and offspring ruled out development by parthenogenesis and hermaphroditism, therefore implicating internal fertilisation by a male stickleback. This extremely rare phenomenon may have been facilitated in this population by an unusual tendency for females to become egg-bound, and suggests that some major transitions may arise almost spontaneously.

## Introduction

Major transitions, involving multifaceted and synergistic changes in multiple traits, pose an evolutionary conundrum because incomplete intermediate forms are unlikely to be functional^1^. Consistent with this, partially developed major transitions are extremely rare^2,3^, and rightly celebrated when discovered^4–6^. The transition from oviparity to retaining developing embryos internally and giving birth to live young involves multiple interrelated changes in physiology and behaviour and as such, has a good claim to being a major transition in the history of life^7–9^. In most animal species, females reproduce oviparously, laying eggs that develop and hatch in the external environment^7^. In many lineages, including teleost fish, some degree of embryo retention has evolved, ranging from ovoviviparity (egg retention) to matrotrophy (placental nourishment and live birth). However, intraspecific variation in such traits is rare (but see Surget-Groba, *et al*.^10^), suggesting that they are under strong selection.

Despite the >35 independent evolutionary origins of ovoviviparity across fish lineages^7^, the huge number of extant egg-laying species (~90% of bony and 43% of cartilaginous fish), and the colossal number of these that must have been examined by fish biologists, records of normally oviparous fish retaining developing embryos are incredibly rare. There are only two previous records of this phenomenon. Multiple specimens of *Hemilepidotus gilberti*, a marine sculpin, were discovered with eyed embryos in the ovaries of spent females, but all embryos died early in development or were deformed and would not have survived post-hatching^11^. A single three-spined stickleback (*Gasterosteus aculeatus*, Linnaeus, 1758, hereafter ‘stickleback’) was also found with well-developed embryos retained in the ovaries^12^, but embryos were not assessed for deformities or signs of life. We document a second occurrence of this phenomenon in stickleback, in which embryos, once removed from the mother, hatched and grew to adulthood without physical abnormalities.

Stickleback are a non-copulatory, oviparous, sexually reproducing species, with a simple XY sex-determining system^13^. Female stickleback are batch spawners^14^ and can become egg-bound (with eggs overripening and hardening in the reproductive tract) if they fail to either spawn or spontaneously release their eggs^15,16^. Retained eggs are lost for reproduction and obstruct the release of further clutches, ultimately killing the female^17,18^. Despite this, egg-bound females are common in some stickleback populations^17,19,20^. The prolonged retention of overripe ovules is considered a non-adaptive ‘accident’^16,18^, but ovule retention is necessary for internal fertilisation, which is an essential step in the transition from oviparity to live-bearing^8^, and thus could be considered a preadaptation to this transition. Preadaptations that arise as non-adaptive by-products of other adaptations, or initially evolve to serve a different function, may facilitate major transitions by subsequently becoming useful in a different role^21–24^.

We discovered an egg-bound marine stickleback in a saline coastal lagoon on North Uist, Scottish Western Isles with ovaries containing well-developed, eyed embryos. This phenomenon has at least three possible explanations, which make distinct, testable predictions: (i) the ova developed by parthenogenesis i.e. by artificial stimulation by heat, chemical or physical shock, in the absence of sperm. Parthenogenesis is common in some animal species^25^, and gynogenesis (development in which the embryo contains only maternal chromosomes due to activation of an egg by a sperm that degenerates without fusing with the egg nucleus) occurs in some fish^26^. Gynogenesis has been stimulated artificially in stickleback^27^, but neither gynogenesis, nor true parthenogenesis have been recorded in natural stickleback populations. This hypothesis requires the parent fish to be genetically and physiologically female and the offspring to be genetically identical to the parent. (ii) Embryos were produced hermaphroditically. Synchronous hermaphroditism, whereby mature individuals possess both functional ovaries and tests, is common in certain teleost species, although all but a single species are outcrossing^28^. *Kryptolebias marmoratus*, a mangrove killifish, is the only known hermaphroditic teleost species that predominantly self-fertilises, and is capable of releasing both fertilised and unfertilised eggs^29,30^. Synchronous hermaphroditism can be chemically induced in stickleback^31^, and an apparently hermaphroditic stickleback population exists in Alaska, from which the single other individual carrying developing embryos was collected^12^, making hermaphroditism an apparently strong possibility. This requires that the parent fish possesses both male and female sex organs, and the offspring carry only alleles present in the parent. (iii) Finally, eggs could have been internally fertilised by the sperm of another stickleback entering the reproductive tract from the external environment, which was the case in *H. gilberti*^11^. This requires the parent fish to be genetically and physiologically female, and the offspring to carry alleles from the father that were absent in the mother. We examined the gonads of the parent fish and performed genetic sex testing, alongside analysis of the allelic composition of parent and offspring to test these hypotheses. To examine the possibility that the presence of the embryos is related to a tendency by fish in the same population to retain eggs, we compared the prevalence of egg-bound gravid females across stickleback populations on North Uist.

## Results

### Analysis of gonads

**Figure 1:**
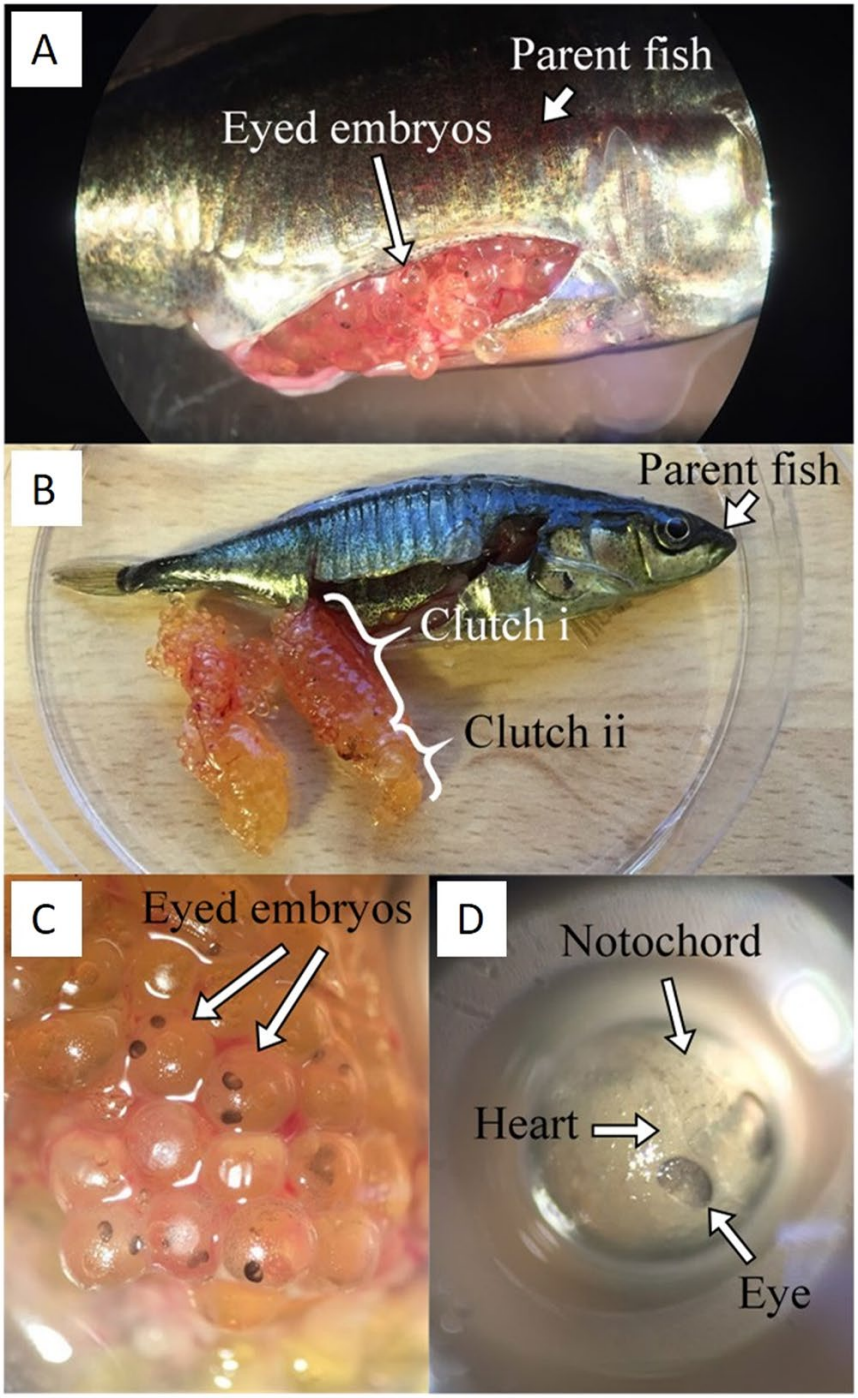
Images of internally developing embryos. (**A**) A female stickleback with ovaries containing internally developing embryos. (**B**) Two developing clutches of eggs in the ovaries. (**C**) A cluster of embryos removed from the ovary, and (**D**) a single embryo removed from the ovary showing developed eyespots, notochord and heart. Photographs were taken by LD.

The parent fish possessed ovaries containing two clutches of eggs, the older (proximal to the cloaca) of which contained many developing embryos (Fig. 1A,B). Embryos were distributed throughout the older clutch and had reached stage 21 of 24 stages of embryonic development in stickleback^32^, Fig. 1C,D. Embryo viability was confirmed by the observation of a heartbeat, and no physical abnormalities, or deformities were observed. Three independent researchers agreed that the gonads of the parent fish contained no testicular tissue (Supplementary Fig. S1), and none was evident elsewhere in the body cavity. Once raised to adulthood, offspring were crossed successfully *in vitro*.

### Sex determination and microsatellites

Microsatellite analysis revealed that the offspring were not genetically identical to the parent fish, or to one another (Table 1). The parent fish was homozygous at four of six microsatellite loci, while two of the offspring were heterozygous at five of six loci (Table 1). Of the 56 offspring, 19 possessed at least one allele that was not present in the parent.

**Table 1:** Genotypes of parent fish and offspring. For sex determining loci, heterozygosity designates males.

Marker	Sex determining	Parent fish			Offspring		
		Homo (1, 1)	Hetero (1, 2)	Homo (2, 2)	Homo (1, 1)	Hetero (1, 2)	Homo (2, 2)
*Idh*	Y	0	0	1	0	15	41
*Gasm6*	Y	0	0	1	0	14	42
*Stn190*	Y	1	0	0	0	13	43
*Stn57*	N	0	1	0	5	5	4
*Stn201*	N	0	1	0	6	5	3
*Stn317*	N	1	0	0	14	0	0

Homo: homozygous, Hetero: heterozygous.

The parent fish was determined to be genetically female by all three sex determining loci (Table 1), while both sexes were present in the offspring (Table 1), but there were more females (42/56 offspring) than males (14/56 offspring, *X*^2^ = 14, *df* = 1, *p* < 0.001).

### Prevalence of egg retention across populations

**Figure 2:**
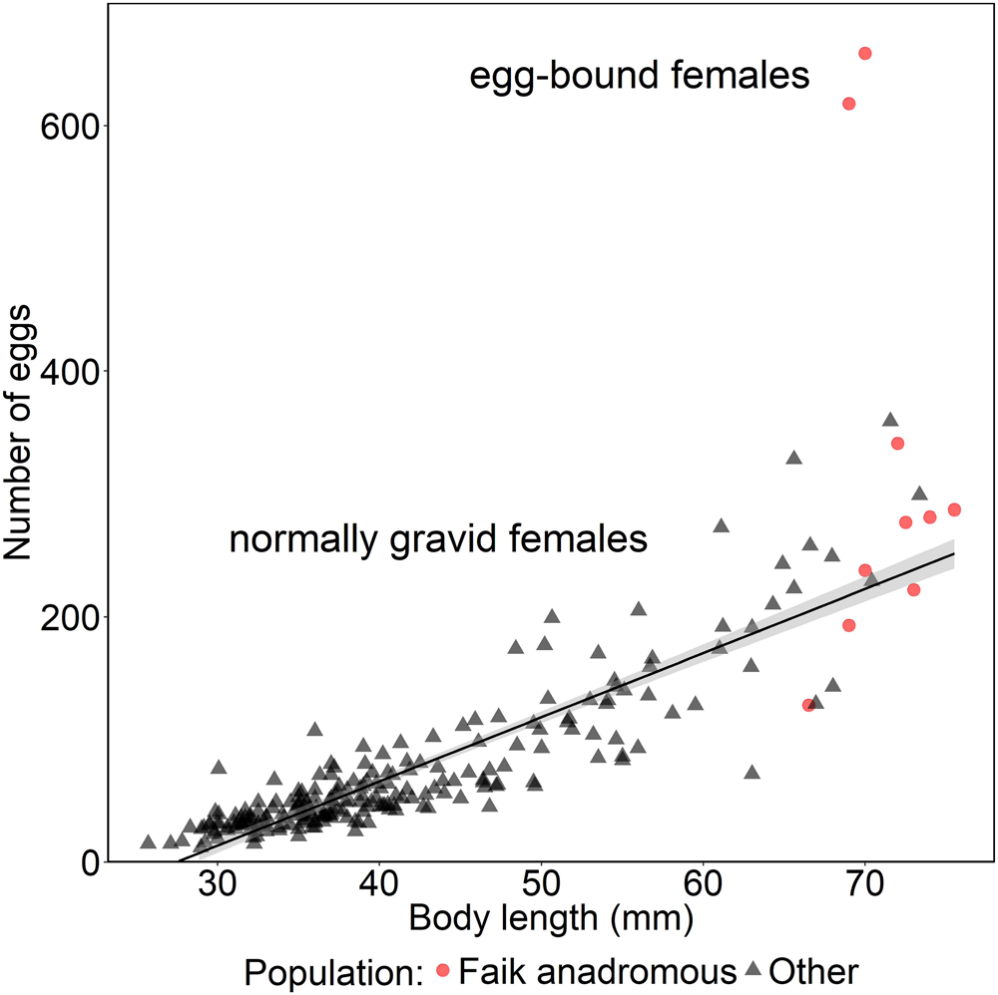
Relationship between body length and number of eggs in normally gravid females. Graph shows the correlation back transformed from the log-log relationship. Red circles: gravid Faik marine females, black triangles: gravid females from 23 other North Uist populations, grey ribbon: ± S.E for normally gravid females.

There was a strong correlation between body length and number of eggs in the ovaries of normally gravid females (r = 0.91, t = 32.24, *df* = 226, *p* < 0.0001, Fig. 2). Egg-bound females, which were found only in Faik, the origin of the parent fish, had almost twice the number of eggs in their ovaries than normally gravid females of a similar size (Fig. 2). Of the 10 gravid marine females in Faik, two were egg-bound (20%), while none of the 208 gravid females from 23 other populations on North Uist were egg-bound (χ^2^ = 24.22, *df* = 1, *p* < 0.0001, Fig. 2).

## Discussion

We identified a clutch of almost fully developed embryos inside the ovaries of a stickleback; a non-copulatory, oviparous species. Embryos had developed normally inside the fish, to the stage of eyeing and body pigmentation, one of the final stages of embryonic development in stickleback^32^, likely corresponding to approximately 5–6 days post-fertilisation, with no visible signs of deformity. This is contrary to the one other known case of internal fertilisation in a non-copulatory fish, where embryos found in the ovaries bore significant deformities^11^. The embryos we removed from the parent fish were viable and fertile, since they hatched, grew to adulthood and were successfully crossed *in vitro* in the lab. However, it is not possible to know whether they would have survived to hatching inside the parent fish, nor whether they would have been able to exit the ovaries, or survive in the wild without the male parental care that is normal in stickleback^33^.

The parent fish was physiologically and genetically female, therefore the embryos cannot have been produced hermaphroditically. Hermaphroditism was implicated as the most likely explanation for the single other record of internally developed embryos in stickleback^12^, however, close inspection of diagrammatic representations of the internal anatomy of stickleback in Greenbank and Nelson^12^, figures 11 and 12, reveals that the authors misinterpreted the normal reproductive anatomy of stickleback as hermaphroditism. We also showed that the offspring we collected were a mix of genetically male and female individuals, and carried alleles for three microsatellite markers that were absent in the mother. This rules out parthenogenesis, and, therefore, the eggs must have been fertilised internally by a separate, male individual. Furthermore, developing embryos were distributed throughout the older clutch of eggs, some well away from the cloaca, indicating that sperm were able to penetrate almost the entirety of the ovaries. The ovarian fluid of female stickleback greatly prolongs sperm motility to facilitate spawning in freshwater^34,35^, and females may prefer to spawn with males whose nests already contain the eggs of other females^36^, thus, we hypothesise that sperm most likely entered the reproductive tract of the parent fish via contact with recently fertilised eggs in a nest.

The fact that the embryos had reached such an advanced stage of development indicates that the ovaries of female stickleback provide a suitable environment for embryonic development. Stickleback embryos acquire all the necessary energy for growth from the yolk provided by the mother within the egg^37^, and although they would not normally develop internally, are capable of modulating their exposure to maternally derived, potentially harmful chemicals^38^. This could explain why embryonic development was not adversely affected by the chemical/hormonal environment of the mother. However, a rich oxygen supply is necessary for proper embryonic development and male stickleback invest heavily in parental care of the eggs, including frequent fanning to increase oxygen delivery^39,40^. Ovoviviparous fish specifically regulate the dissolved oxygen content in ovarian fluid according to embryonic requirements^41^, but whether this is possible in stickleback is not known. Nonetheless, the normal development of embryos inside the ovaries in this case indicates that there must have been sufficient oxygen for normal development, and that both female stickleback and their embryos already possess many of the preadaptations necessary for embryos to develop within the ovaries.

Given the extensive research that has been conducted on stickleback, a model organism^42–44^, and oviparous fish in general, the fact that internal embryonic development has only been recorded once before in stickleback^12^, and only once more across all other normally oviparous fish^11^, suggests that it is extremely uncommon. The population to which the parent fish belonged had an increased tendency towards prolonged egg retention (20% of anadromous females in Faik were egg-bound, while no egg-bound females were found across 23 other populations), which could increase the likelihood of accidental internal fertilisation during failed egg-laying attempts. However, egg-bound females have been observed in other stickleback populations^14,17^, and oviparous fish species^18,45–47^, with no record of internal embryonic development. Nonetheless, prolonged egg retention could be a preadaptation that facilitates the transition to internal embryonic development. We cannot know whether such isolated incidence of the internal retention of developing embryos indicates the nascent evolution of a transition to live-bearing, but they do suggest that such transitions are possible and can arise more or less spontaneously.

## Methods

### Fish collection, dissection and egg husbandry

As part of an ongoing program to breed stickleback and collect data on life history, gravid females have been sampled over several years (2007–2018) from many lochs and coastal lagoons on the Scottish Hebridean island of North Uist using un-baited minnow traps (Gee traps, Dynamic Aqua, Vancouver) set overnight. A single brackish lagoon, Fairy Knoll (“Faik”, 57°38′7′N; 7°12′54′W), appeared to have a consistently high abundance of egg-bound females with ~1–3 being found each year. When suspected egg-bound females were caught, they were euthanized and dissected as below and always found to be egg-bound with unfertilised over-ripe ovules. As a result of the prevalence of egg-bound females, breeding females in Faik were monitored in an ad-hoc way for a number of years and on 24^th^ April 2015 a gravid female marine stickleback, displaying the classic ‘berried’ abdomen of an egg-bound fish^17^, was caught in this lagoon. The fish was transported to the laboratory in aerated loch water and euthanized on arrival by overdose of MS222 anaesthesia, followed by destruction of the brain, in accordance with Schedule One of UK Home Office regulations. Following dissection, eggs containing developing embryos were identified inside the intact peritoneal membranes surrounding the ovaries. Eggs, and all internal organs were thoroughly examined, particularly for the presence of testicular tissue, using a light microscope. Eggs containing developing embryos were kept in chilled 1% saline solution and transported to aquaria at the University of Nottingham. Between four and seven days following the dissection, 56 fry hatched and were raised to adulthood in the lab. Adult offspring were subsequently crossed *in vitro* to determine fertility. Following the discovery of the stickleback containing fertilised embryos, further sampling efforts were made in Faik between April and May of 2015, 2016 and 2017 to find more females in the same condition, but only females egg-bound with over-ripe ovules were found. All methods were carried out in accordance with the Animals (Scientific Procedures) Act 1986 and all experimental protocols were approved by the University of Nottingham Animal Welfare and Ethical Review Body (AWERB), and conducted by licenced personnel under the UK Home Office Project Licence 30/3415.

### Sex determination and microsatellite analysis

The gonads of the parent fish were fixed, stained, sectioned (see Supplementary Material for detailed methodology), and examined thoroughly for the presence of testicular tissue by three independent researchers to determine physiological sex. Genetic sex determination was conducted on the parent fish and 56 offspring by PCR amplification (see Supplementary Table S1 for PCR primers) of three loci: *Idh*, *Gasm6* and *Stn190*, which, combined, produce an accurate determination of sex^48^. To further assess whether a second individual contributed to the genetic make-up of the offspring three additional sets of microsatellite loci (*Stn57*, *Stn201* and *Stn317*) were also analysed for the parent fish and 14 randomly selected offspring.

DNA was collected by running a single sterile swab over both flanks of each fish and extracted using a proteinase K extraction procedure. Swab heads containing DNA samples were removed and placed on ice in individual Eppendorf tubes prior to DNA extraction. DNA was extracted using Viagen DirectPCR Lysis Reagent (Mouse Tail), following the manufacturers standard protocol with the following modifications. 270 µl of DirectPCR Lysis Reagent and 30 µl proteinase K were added to each swab and samples were incubated for 5 hours at 55 °C whilst rotating at 250 rpm. The swab head was removed from each tube, and the solution then incubated at 85 °C for 45 minutes before cooling to room temperature. All PCR reactions were carried out in 20 µl volumes containing 6 µl nuclease-free H_2_0, 10 µL 2X Biomix red reaction mix (Bioline), 1 µl of both forward and reverse primers (10 µM) and 2 µl of template DNA (approximately 20 ng). For all PCRs, thermocycling was set up as follows: Initial denaturation at 96 °C for two minutes, followed by 35 cycles of denaturation at 96 °C for 30 seconds, annealing at 56 °C for 30 seconds and extension at 70 °C for 30 seconds, followed by a final extension at 70 °C for two minutes. To score genotypes, PCR products were electrophoresed on a 4% agarose gel run at 100 V for one hour, alongside a 100 bp DNA ladder. For sex testing primers the presence of a single band indicated a female and the presence of a double band indicated a male. On the few occasions where the result of one primer pair differed from the others, the sex of the fish was assumed to be that of which two out of the three pairs of primers agreed. For all six microsatellite loci the occurrence of alleles in the offspring that are not present in the mother indicated that a second individual contributed to the genetic make-up of the offspring.

### Analysis of egg-bound females

During April-May of 2007–2008 and 2010–2011, 230 gravid female stickleback were collected from 24 populations on North Uist (Supplementary Table S2). Females were euthanized as above, measured for standard length and dissected. Ovaries were removed and all eggs were counted and assessed for signs of being overripe^17^.

### Statistical analysis

Statistical analyses were carried out in R version 3.4.4^49^. To test for a correlation between body length and number of eggs in normally gravid females (egg-bound females were excluded because of their substantial increase in number of eggs), both variables were log transformed and a t-test based on Pearson’s correlation was used. To test whether the sex ratio in the offspring differed from an expected ratio of 50:50, and to test for differences in the prevalence of egg-bound females in the marine population in Faik, compared with all other populations sampled on North Uist combined (Supplementary Table S2), chi-squared tests were used.

## Supplementary information

Supplementary material

Dataset 1

Dataset 2

## Data Availability

Egg count and microsatellite data are provided as supplementary material.
