# Supplementary material for: Internal embryonic development in a non-copulatory, egg-laying teleost, the three-spined stickleback, Gasterosteus aculeatus
Source: Sci Rep. 2019 Feb 20;9:2395. doi: 10.1038/s41598-019-38584-w (PMC6382768; doi:10.1038/s41598-019-38584-w)
Supplement: Supplementary file 1 — Supplementary material [file 41598_2019_38584_MOESM1_ESM.docx]

**Supplementary material for: Internal embryonic development in a non-copulatory, egg-laying teleost, the three-spined stickleback, *Gasterosteus aculeatus***

Laura L. Dean^1^*, Shaun Robertson^1^, Muayad Mahmud^1^ and Andrew D.C. MacColl^1^

^1^School of Life Sciences, University of Nottingham, NG7 2RD, UK

*laura.dean@nottingham.ac.uk

### Additional methodological and data details

Gonadal tissue was rehydrated by the sequential dilution of ethanol from 70% to 0%, using deionised water. Gonadal tissues were then fixed in 10% neutral buffered formalin, dehydrated in graded alcohol-xylene and embedded in paraffin wax using a Leica TP1020 Tissue Processor. Sections of tissue (8µm thick) were taken at 500μm intervals throughout the ovarian tissue that remained following the removal of developing embryos from the ovaries using a Slee Cut 4060 Microtome and stained using Haematoxylin-Eosin before being mounted on glass microscope slides using DPX. Sections from the parent fish were compared to those from three male and three female individuals (sexed both genetically and morphologically) from a nearby waterbody, Loch a Chadha Ruaidh (Chru, 57°35'37"N; 7°11'44"W).

Supplementary Table S1

PCR primers for sex determination and microsatellite analysis.

| **Primer** | **Sequence** | **Source**  **choice)** |
| --- | --- | --- |
| ldh_F | 5’ GGGACGAGCAAGATTTATTG 3’ | (16) |
| ldh_R | 5’ TTATCGTTAGCCAGGAGATGG 3’ | (16) |
| Gasm6_F | 5’ GATTAAAGGAACCAGAGGGG 3’ | (48) |
| Gasm6_R | 5’ GGAACTGGAATTTTGAGGGT 3’ | (48) |
| Stn190_F | 5’ CGATAATGCACGACAATCTCC 3’ | (49) |
| Stn190_R | 5’ AACACATGAGCGTTATGGC 3’ | (49) |
| Stn57_F | 5’ GATGGTGCCCATAAGACTCG 3’ | (49) |
| Stn57_R | 5’ CATGTGTGGATGAAGGATGC 3’ | (49) |
| Stn201_F | 5’ TCACTTCACAGGGACAATGG 3’ | (49) |
| Stn201_R | 5’ ACTGCTGGAGGATGAAATGG 3’ | (49) |
| Stn317_F | 5’ CAGGATGAAATGAAGGTCTGG 3’ | (49) |
| Stn317_R | 5’ TGTGGACTTTCAGATGAGCG 3’ | (49) |

Primers are identified by the name of the loci followed by ‘F’ for forward primers and ‘R’ for reverse primers. Source describes the researchers by whom primers were originally developed.

Supplementary Table S2

Stickleback sampling locations.

| **Loch name** | **Loch ID** | **Population** | **N** | **Salinity**  **choice)** | **Location** |
| --- | --- | --- | --- | --- | --- |
| Abhainn Malacleit | Abma | Resident | 6 | Fw | 57°38'37"N; 7°21'55"W |
| A'Charra | Acha | Resident | 1 | Fw | 57°35'39"N; 7°23'18"W |
| Mhic A'Roin | Aroi | Resident | 2 | Fw | 57°35'40"N; 7°25'52"W |
| A Bharpa | Bhar | Resident | 11 | Fw | 57°34'16"N; 7°18'8"W |
| Bheireagvat | Bhei | Resident | 4 | Fw | 57°37'46"N; 7°13'45"W |
| Na Buaile | Buai | Resident | 19 | Fw | 57°38′48"N; 7°11′48"W |
| A Chadha Ruaidh | Chru | Resident  Resident | 7 | Fw | 57°35'37"N; 7°11'44"W |
| An Daimh | Daim | Resident | 9 | Fw | 57°35'32"N; 7°12'30"W |
| Dubhasairidh | Dubh | Resident | 6 | Fw | 57°34′54"N; 7°24′12"W |
| Eisiadar | Eisi | Resident | 3 | Fw | 57°37'50"N; 7°21'8"W |
| Fairy Knoll | Faik | Resident | 12 | Brackish | 57°38'7"N; 7°12'54"W |
|  |  | Marine | 10 |  |  |
| Fhaing Buidhe | Fhai | Resident | 12 | Fw | 57°34'6"N; 7°22'45"W |
| Mhic Gille Bhride | Gill | Resident | 9 | Fw | 57°36'4"N; °24'37"W |
| Hosta | Host | Resident | 21 | Fw | 57°37′40"N; 7°29′18"W |
| Leodasay | Leod | Marine | 2 | Brackish | 57°32'39"N; 7°20'8"W |
| Magarlan | Maga | Resident | 12 | Fw | 57°36′10"N; 7°28′54"W |
| Na Maighdein | Maig | Resident | 12 | Fw | 57°35'44"N; 7°12'15"W |
| Na Moracha | Mora | Resident | 5 | Fw | 57°34'29"N; 7°16'31"W |
| Ob nan Stearnain | Obse | Resident | 2 | Brackish | 57°36'6"N; 7°10'22"W |
|  |  | Marine | 9 |  |  |
| Na Reivil | Reiv | Resident | 14 | Fw | 57°36'39"N; 7°30'50"W |
| Scadavay (South) | Scad | Resident | 18 | Fw | 57°35′6"N; 7°14′10"W |
| Tormasad | Torm | Resident | 24 | Fw | 57°33'46"N; 7°19'1"W |

Lochs on North Uist, Scottish Western Isles, from which gravid female stickleback were collected. N = total sample size of gravid stickleback. Fw: freshwater. Location is given by latitude followed by longitude.

### Additional imagery

| 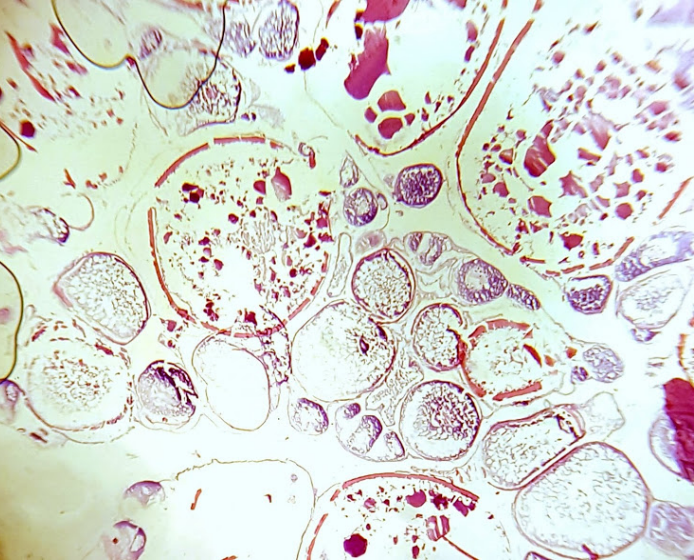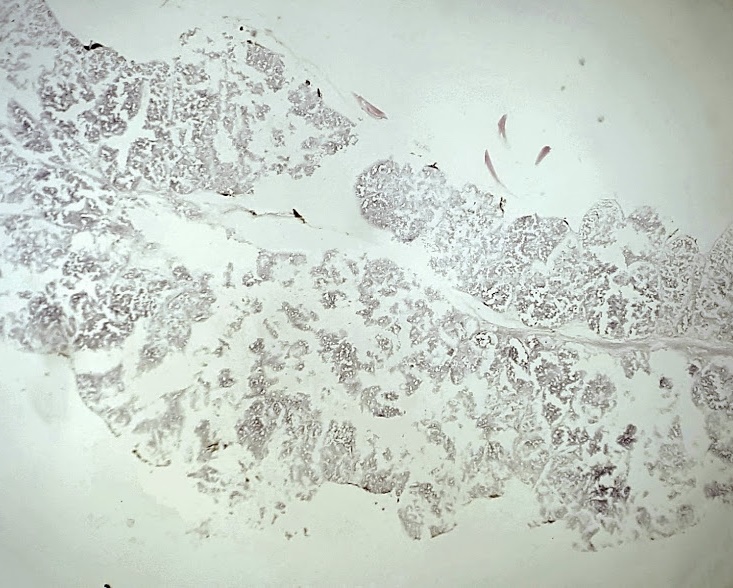  *  *  A  B  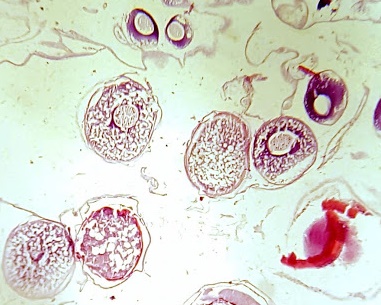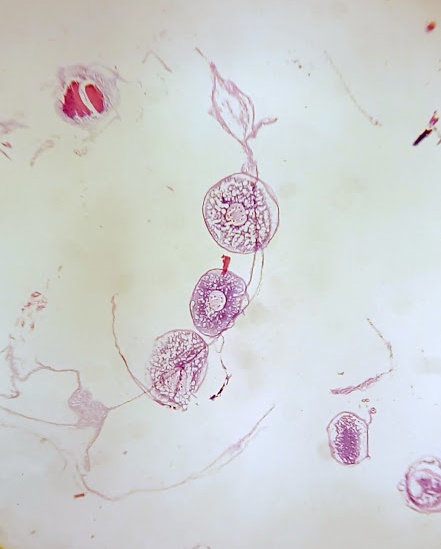  *  *  D  C |
| --- |

Supplementary Figure S1 Cross sections of gonadal tissue.

Cross sections of (A) ovarian and (B) testicular tissue of confirmed genetic female and male threespine stickleback respectively. (C & D) Cross sections of the gonadal tissue of the parent fish carrying developed embryos. Black bars indicate 200µm, arrows show mature oocytes post vitellogenesis and stars indicate immature follicles.
